# Hydrogelation from Self-Assembled and Scaled-Down Chitin Nanofibers by the Modification of Highly Polar Substituents

**DOI:** 10.3390/gels9060432

**Published:** 2023-05-23

**Authors:** Jun-ichi Kadokawa

**Affiliations:** Graduate School of Science and Engineering, Kagoshima University, 1-21-40 Korimoto, Kagoshima 890-0065, Japan; kadokawa@eng.kagoshima-u.ac.jp; Tel.: +81-99-285-7743

**Keywords:** chitin, highly polar substituent, hydrogelation, nanofiber, network structure, physical crosslinking

## Abstract

Chitin nanofibers (ChNFs) with a bundle structure were fabricated via regenerative self-assembly at the nanoscale from a chitin ion gel with an ionic liquid using methanol. Furthermore, the bundles were disentangled by partial deacetylation under alkaline conditions, followed by cationization and electrostatic repulsion in aqueous acetic acid to obtain thinner nanofibers called scaled-down ChNFs. This review presents a method for hydrogelation from self-assembled and scaled-down ChNFs by modifying the highly polar substituents on ChNFs. The modification was carried out by the reaction of amino groups on ChNFs, which were generated by partial deacetylation, with reactive substituent candidates such as poly(2-oxazoline)s with electrophilic living propagating ends and mono- and oligosaccharides with hemiacetallic reducing ends. The substituents contributed to the formation of network structures from ChNFs in highly polar dispersed media, such as water, to produce hydrogels. Moreover, after the modification of the maltooligosaccharide primers on ChNFs, glucan phosphorylase-catalyzed enzymatic polymerization was performed from the primer chain ends to elongate the amylosic graft chains on ChNFs. The amylosic graft chains formed double helices between ChNFs, which acted as physical crosslinking points to construct network structures, giving rise to hydrogels.

## 1. Introduction

**Figure 1:**
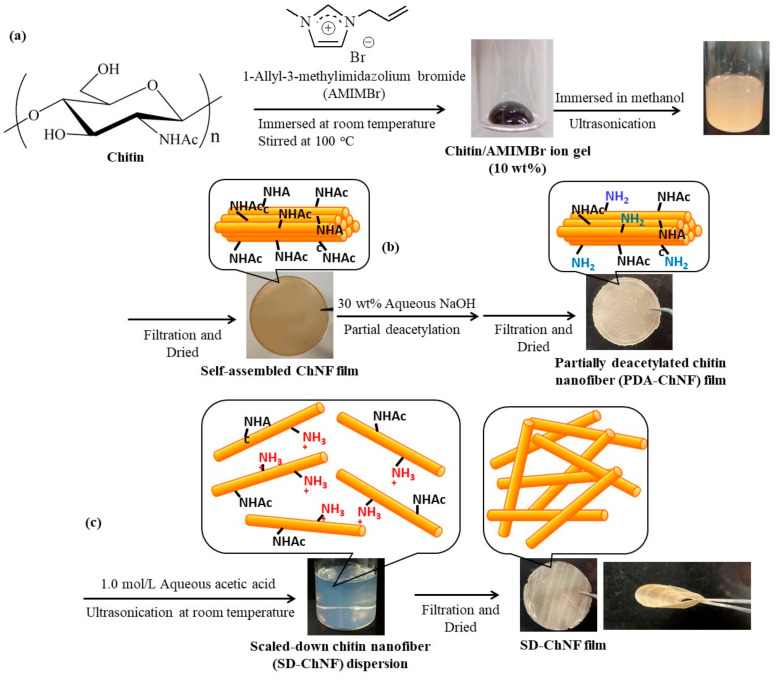
Procedures for the preparation of (**a**) a self-assembled chitin nanofiber (ChNF) film, (**b**) partially deacetylated (PDA)-ChNF film, (**c**) scaled-down (SD)-ChNF film.

Polysaccharides, which are natural polymers composed of monosaccharide residues linked through glycosidic linkages, are widely present on Earth and are regarded as structural and energy storing materials [1,2,3]. Chitin, comprising main chains of β(1→4)-linked *N*-acetyl-d-glucosamine (GlcNAc) residues (Figure 1a), is one of the most abundantly distributed polysaccharides and is prevalent in the exoskeletons of crustaceans [4,5,6]. Despite its enormous annual bioproduction, chitin is mostly unutilized as a biomass resource because of its poor feasibility and processability. This is because of its robust crystalline structure and extended fibrous chain packing, which is composed of numerous intra- and intermolecular hydrogen bonds [7,8,9,10,11].

Recently, the construction of controlled polymeric assemblies at the nanoscale (e.g., nanofibers, nanocrystals, and nanowhiskers) has been found to be an efficient approach for the functionalization of chitin [12,13,14], owing to the remarkable properties of bio-based nanomaterials, such as their lightweight character, nanosheet formability, high tensile strength, low thermal expansion coefficient, and biocompatibility [15,16,17,18,19,20,21]. Previously, two types of approaches for providing such nanochitins have been developed: a top-down approach, where natural chitin sources break down to the nanoscale [12,13,14], and a bottom-up approach, where chitin chains self-assemble regeneratively at the nanoscale [22,23,24]. Moreover, nanochitins have been employed in biomedical applications, such as wound healing and bone regenerative engineering [25,26,27,28].

Based on the bottom-up approach, the author’s research group has already reported a facile method to obtain chitin nanofibers (ChNFs) with a length of several hundred nanometers and a width of approximately 20–60 nm through regeneration from an ion gel using methanol (Figure 1a) [29,30]; the ion gel is facilely formed from a mixture of chitin with an ionic liquid (IL), 1-allyl-3-methylimidazolium bromide (AMIMBr), by heating at 100 °C. This result was based on our previous finding that AMIMBr dissolves or swells chitin via simple operations [31]. The resulting ChNF/methanol dispersion was subjected to suction filtration for isolation, yielding a ChNF film with a heavily entangled nanofiber assembly.

Moreover, such self-assembled ChNFs comprised a bundle-like structure hierarchically fabricated by the assembly of thinner fibrils [32]. The author’s research group successfully developed a method for the disentanglement of bundles, including partial deacetylation and cationization, to obtain individual thinner nanofibril dispersions in aqueous acetic acid [33]. The isolation of thin fibrils from the resulting dispersion via suction filtration formed a film with a heavily condensed morphology, which exhibited superior flexibility compared to that of the partially deacetylated (PDA)-ChNF film. The thinner nanofibers were accordingly named ‘scaled-down ChNFs (SD-ChNFs)’ (Figure 1b,c).

Hydrogels are one of the most useful soft materials obtained by constructing nanoscale network structures from polymeric substrates. A hydrogel is a biphasic material comprising porous, permeable solids with a three-dimensional network structure and a large volume of dispersed aqueous media. Polymeric hydrogels are classified according to the types of crosslinks used for the formation of networks from polymers. Chemical hydrogels have covalent crosslinking bonds, whereas physical hydrogels have non-covalent bonds, that is, physical interactions. The hydrogelation of ChNFs was successfully achieved by the efficient formation of ChNF networks, mainly by physical crosslinking. For example, the treatment of ChNFs with aqueous acidic and alkaline solutions, such as aqueous NaOH, produces network structures that give rise to chitin hydrogels [34,35,36]. The physical crosslinking of ChNFs with other components has also been performed to produce hydrogels [37].

**Figure 2:**
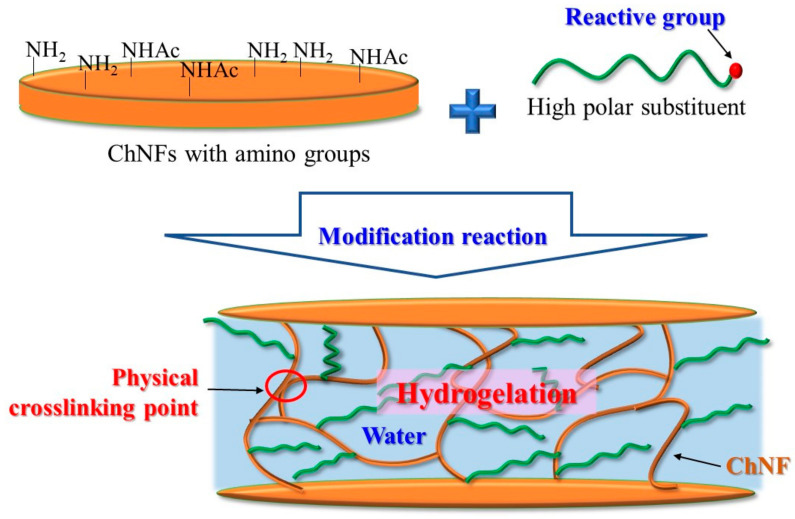
A schematic image for the formation of the network structure with physical crosslinking points by modification of highly polar substituents on ChNFs.

The author’s research group achieved physical hydrogelation from the abovementioned PDA- and SD-ChNFs by appropriately modifying the highly polar substituents, where the network structures were gradually formed by ChNF crosslinking during the modification reactions (Figure 2) [38]. The modification was successfully performed by the reaction of the amino groups on the ChNFs with the reactive groups in the substituents. For example, when well-known biocompatible polymers, that is, poly(2-oxazoline)s (POxs), were grafted on ChNFs by the ‘grafting-to’ approach, gelation progressed to form not only hydrogels with aqueous disperse media but also organogels with organic disperse media according to the structural changes to the grafted POxs [39,40]. Moreover, modification of mono- and oligosaccharides on PDA-ChNF films by reductive amination was performed to form physical hydrogels [41]. After modifying the oligosaccharide primers on the PDA-ChNF films, enzymatic polymerization was performed at the primer chain ends to produce amylose-grafted ChNFs. The products also formed hydrogels by physical crosslinking based on a double helical formation from elongated amyloid chains [42]. In this review article, these physical hydrogelation methods from self-assembled and SD-ChNFs by modifying highly polar polymers and mono-, oligo-, and polysaccharides are discussed.

## 2. Fabrication of Self-Assembled SD-ChNFs

Self-assembled ChNFs were prepared based on a bottom-up approach by regeneration from ion gels with an IL, that is, AMIMBr, using methanol (Figure 1a) [29,30]. The ILs were identified as molten salts with melting points below the boiling point of water. ILs are well-known, powerful solvents for polysaccharides [22,24,31,43,44,45,46,47], and Rogers et al. first reported the dissolution of cellulose in the ionic liquid 1-butyl-3-methylimidazolium chloride [48]. Some ILs, including AMIMBr, have been found to dissolve or swell up chitin [24]. The slow regeneration from the 6.5–10.7 wt% chitin ion gels with AMIMBr, which was performed by immersion in methanol at room temperature for 24 h, followed by ultrasonication, produced ChNF dispersions. A ChNF film was then fabricated by isolating the resulting self-assembled ChNFs via suction filtration of the dispersion. The SEM image of the resulting film reveals a heavily entangled nanofiber morphology, in which the entangled nanostructures from the ChNFs likely contributed to the formation of the film.

As mentioned above, the self-assembled ChNFs were found to form bundles upon hierarchical assembly from thin fibrils, which were supported by the TEM image of the ChNF/methanol dispersion before suction filtration [32]. In a subsequent study, accordingly, an attempt was made to obtain thinner individual nanofibers [33]. The self-assembled ChNF film was first treated with 30 wt% aqueous NaOH for deacetylation to partially generate amino groups on the PDA-ChNFs (Figure 1b), which could be converted to the cationic form under acidic conditions. Therefore, treatment of the resulting PDA-CNF film with 1.0 mol/L aqueous acetic acid caused electrostatic repulsion that disentangled the ChNFs, giving rise to a dispersion of the individual SD-ChNFs (Figure 1c). The isolation of SD-ChNFs via suction filtration of the dispersion resulted in a highly flexible film that was easily bent and twisted. In subsequent studies, the resulting self-assembled and SD-ChNFs were modified with highly polar substituents via the appropriate reactions to occur hydrogelation.

## 3. Hydrogelation from Self-Assembled ChNFs by Grafting Poly(2-oxazoline)s

**Figure 3:**
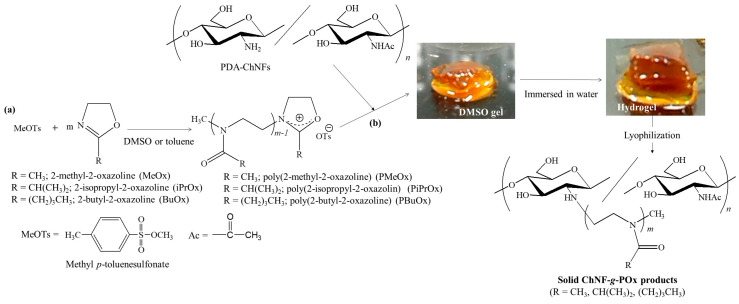
(**a**) Cationic ring-opening polymerizations of 2-alkyl-2-oxazoline (Oxs) and (**b**) grafting poly(2-alkyl-2-oxazoline)s (POxs) onto PDA-ChNF film (adapted with permission from Ref. [40]. Copyright 2021, Elsevier).

**Figure 4:**
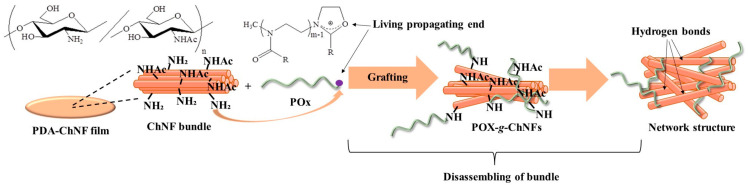
The proposed process for the formation of the network structure from chitin nanofibers (ChNFs) by grafting poly(2-oxazoline) (POx) for gelation (adapted with permission from Ref. [40]. Copyright 2021, Elsevier).

The author’s research group reported the grafting of POxs onto the PDA-ChNF film according to the ‘grafting-to’ approach, which proceeds by the reaction of living propagating ends of POxs with amino groups on the film in DMSO [39,40]. The family of POxs is particularly noteworthy because it has been extensively studied as a family of versatile biocompatible polymers that can be used to produce environmentally friendly materials [49,50,51,52,53]. Moreover, because POxs can be prepared by living cationic ring-opening polymerization of 2-oxazolines (Oxs) as monomers with electrophilic initiators, such as methyl *p*-toluenesulfonate (MeOTs, Figure 3a), living propagating ends show an electrophilic nature and react efficiently with nucleophiles, such as amino groups, to covalently attach the desired groups and substrates at the POx chain end [54,55,56,57,58,59]. Interestingly, the substituents at position 2 on the Ox rings strongly affect the POx properties [58,59,60]; for example, the carbon chain lengths in the substituents determine the hydrophobic and hydrophilic properties of the POxs. When poly(2-methyl-2-oxazazoline) (PMeOx), with strong hydrophilicity and high polarity, was used for the grafting reaction with the PDA-ChNF film in DMSO, an organogel with DMSO gradually formed (Figure 3b) [39]. The resulting organogel was converted into a hydrogel by exchanging DMSO with water. During the grafting reaction, the highly polar PMeOx graft chains drew DMSO into the film, leading to the disentanglement of ChNFs. Further disassembly of ChNF bundles occurred partially by the absorption of DMSO into the bundles, resulting in the formation of a network structure with ChNF crosslinking from the thin fibers for gelation (Figure 4). In the product, the thin fibers formed multipoint interactions via hydrogen bonds on the surfaces, which acted as physical crosslinking points, giving rise to a network structure.

Poly(2-isopropyl-2-oxazoline) (PiPrOx) and poly(2-butyl-2-oxazoline) (PBuOx) with more hydrophobicity and lower polarity than PMeOx were also grafted onto PDA-ChNF films, according to the same ‘grafting-to’ procedure as above (Figure 3b) [40]. All the products formed gels with highly polar dispersed media, such as hydrogels with water and organogels with DMSO. In addition, the ChNF-*g*-PBuOx product could also form organogels with relatively nonpolar dispersed media. Both the polar amino groups and low-polarity PBuOx chains present in ChNF-*g*-PBuOx probably induce gelation in a wide range of dispersed media.

## 4. Hydrogelation from Self-Assembled SD-ChNFs by the Modification of Mono- and Oligosaccharides

**Figure 5:**
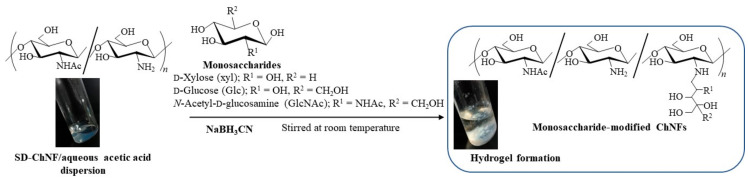
Reductive amination of monosaccharide residues on SD-ChNFs.

**Figure 6:**
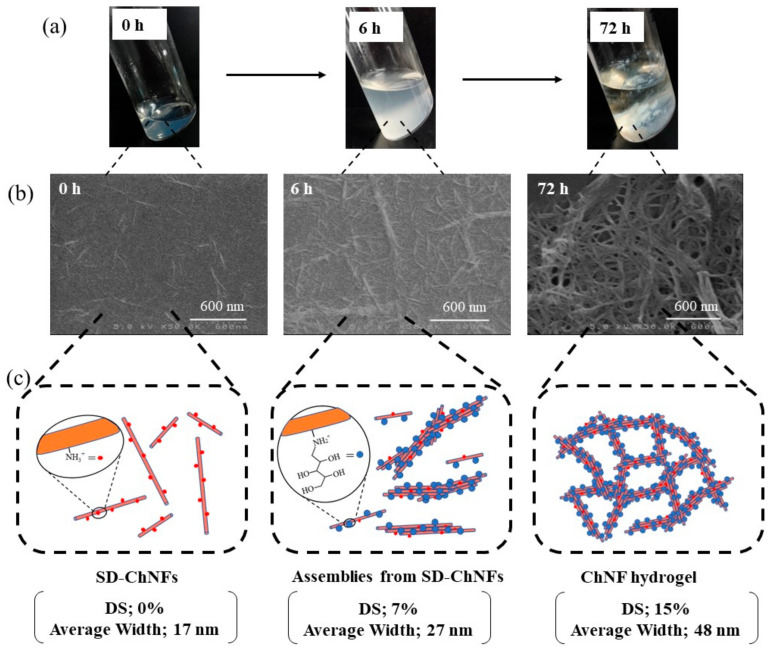
(**a**) Photographs of reductive amination mixtures, (**b**) SEM images of samples spin-coated from reductive amination mixtures from d-xylose and SD-ChNFs in accordance with reaction times, and (**c**) the plausible mechanism for hydrogelation via the formation of the network structure (adapted with permission from Ref. [41]. Copyright 2022, The Society of Fiber Science and Technology, Japan).

The modification of monosaccharide residues (d-xylose (Xyl), d-glucose (Glc), and GlcNAc) with SD-ChNFs by reductive amination gave rise to network structures, leading to the formation of hydrogels [41]. Reductive amination is a well-known reaction for amino derivatization at the hemiacetallic-reducing end of saccharide chains using primary amines via an imine intermediate [61]. Accordingly, the reductive amination of Xyl on SD-ChNFs was carried out in a mixture of Xyl and the reducing agent, NaBH_3_CN, with an SD-ChNF/aqueous acetic acid dispersion (200 equiv. with amino groups) at room temperature under stirring to obtain Xyl-modified ChNFs (Figure 5). With a prolonged reaction time, the mixture gradually became turbid. Subsequently, after 72 h, a hydrogel was formed, as shown in Figure 6a, right. The SEM image of the lyophilized hydrogel sample illustrates the network morphology at the nanoscale (Figure 6b, right). Hydrogels were also formed by the reductive amination of Glc and GlcNAc on SD-ChNFs under the same conditions.

To propose a mechanism for the formation of hydrogels in the present system, the morphological changes during the reductive amination of Xyl groups on SD-ChNFs were investigated. As shown in Figure 6a, left and center, the gradual formation of aggregates was observed in the reductive amination mixtures with increasing reaction times, which were probably constructed by the assembly of the SD-ChNFs. The SEM image of the sample prepared by spin-coating the mixture, obtained at a reaction time of 6 h, showed a thicker nanofiber morphology (Figure 6b, center) than the parent SD-ChNFs (Figure 6b, left). A hydrogel was formed in the mixture after 72 h (Figure 6a, right). The SEM image of the sample prepared by spin-coating the gel-like aggregates shows a network morphology at the nanoscale (Figure 6b, right). Overall, the average nanofiber width increased with increasing DS values of the Xyl residues according to the reaction time, as shown in Figure 6.

Based on the above investigation, the following mechanism was proposed for the formation of hydrogels by the reductive amination of monosaccharide residues on SD-ChNFs (Figure 6). The disentanglement of the bundle assembly of PDA-ChNFs occurred by cationization and strong electrostatic repulsion in 1.0 mol/L aqueous acetic acid to produce SD-ChNFs. The Xyl modification gradually weakened the electrostatic repulsion among SD-ChNFs, resulting in their assembly. With increasing reaction time, a network structure at the nanoscale was hierarchically formed by further interactions of the assembled SD-ChNFs, resulting in the formation of a hydrogel in the mixture.

**Figure 7:**
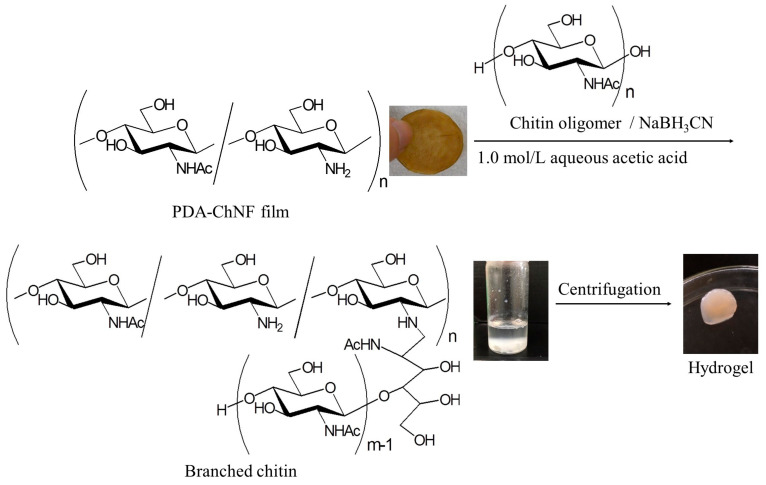
A reductive amination of a chitin oligomer on SD-ChNFs.

The reductive amination approach was extended to the fabrication of branched chitin structures using PDA-ChNFs and chitin oligomers (commercially available; average degree of polymerization = 1.5). When reductive amination of the chitin oligomer on the PDA-ChNF film (50 equiv. with amino groups) was performed in the presence of NaBH_3_CN as the reducing agent (50 equiv. with amino groups) in 1.0 mol/L aqueous acetic acid at 40 °C for 1 h with stirring, gel-like aggregates were formed in the reaction mixture, which were isolated by centrifugation (Figure 7). A gelation procedure for the resulting branched chitin, similar to that for POxs, was proposed.

## 5. Hydrogelation by Enzymatic Grafting of Amylose on PDA-ChNFs

Amylose, a representative natural polysaccharide, is a component of starch [1]. Because amylose forms a left-handed helical conformation owing to its glucan chain (Glc polymer) structure linked through α(1→4)-glycosidic linkages, it readily forms a double-helical assembly [62,63]. Pure amylose, solely composed of α(1→4)-linked Glc units, is prepared by the glucan phosphorylase (GP)-catalyzed enzymatic polymerization of an-d-glucose 1-phosphate (Glc-1-P) monomer, initiated from a maltooligosaccharide (α(1→4)-linked Glc oligomer) primer [64,65,66,67,68,69,70]. As polymerization strictly propagates from the non-reducing end of the primer, the other end (i.e., the reducing end) does not participate in the reaction. Accordingly, even when the reducing end of the primer is covalently linked to other polymers by chemical reactions, enzymatic polymerization occurs from the non-reducing primer ends modified on the polymeric main chain to provide amylose-grafted polymeric materials (chemoenzymatic approach) [71,72,73].

**Figure 8:**
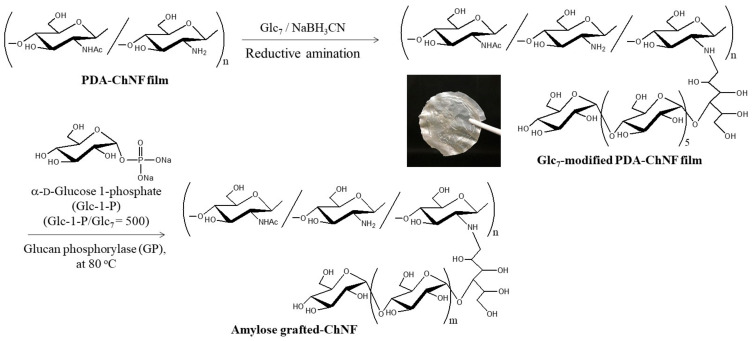
A procedure for the preparation of an amylose-grafted self-assembled chitin nanofiber (ChNF) by a chemoenzymatic approach (adapted with permission from Ref. [42]. Copyright 2018, American Chemical Society).

**Figure 9:**
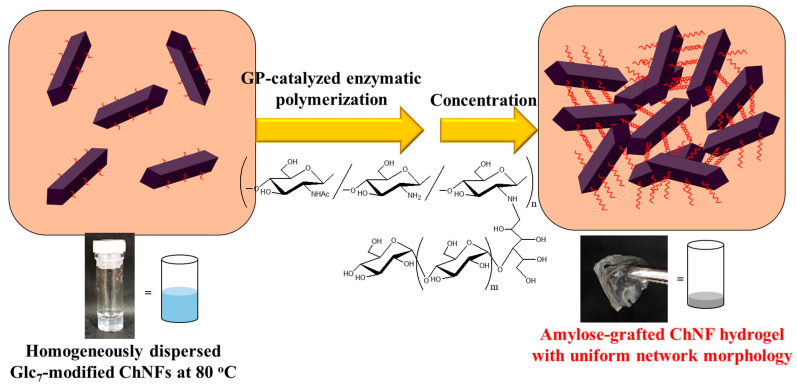
A schematic image for the formation of hydrogels by the construction of a network structure via GP-catalyzed enzymatic polymerization from a Glc_7_ primer on ChNFs.

The author’s research group synthesized amylose-grafted ChNFs using a chemoenzymatic method (Figure 8) [42]. Maltooligosaccharide (maltoheptaose, Glc_7_) primers were first modified on the PDA-ChNF film by reductive amination, similar to the approach described in Section 4, to obtain a Glc_7_-modified ChNF film. After the resulting Glc_7_-modified ChNF film was dispersed in an aqueous sodium acetate buffer by ultrasonication, Glc-1-P (500 equiv. with the non-reducing end of the primer) and GP were added to the dispersion, and the mixture was maintained at 80 °C for 6 h with stirring in a closed vessel for enzymatic polymerization. The resulting mixture was concentrated by heating further at 80 °C for 3 h in an opened vessel. It gradually became viscous and subsequently turned into a hydrogel. The XRD profile of the lyophilized form of the hydrogel exhibited a diffraction pattern ascribed to the amylosic double helical crystalline structure, accompanied by a peak assigned to the chitin crystal. This observation strongly indicates that physical crosslinking from amylosic double helices among ChNFs induced the formation of a network structure, which was significant for the hydrogelation behavior of the present material (Figure 9). The Glc_7_-modified ChNFs dispersed well in a sodium acetate aqueous buffer at 80 °C, and a uniform network was fabricated by the formation of the double helixes from the amylosic chains, enzymatically elongated from the Glc_7_ primer chains on ChNFs, giving rise to hydrogelation.

## 6. Conclusions

This review showed that the modification of highly polar substituents, such as POxs and mono-, oligo-, and polysaccharides, on ChNFs, is an efficient approach to physically induce hydrogelation with network structures. By modifying the reactions of the amino groups on ChNFs with appropriate reactive groups in the substituent candidates, physical crosslinking points from ChNF chains or enzymatically elongated amylosic graft chains on ChNFs are constructed to form network structures. The present hydrogels are entirely composed of biodegradable or biocompatible components, such as saccharides and POxs, and exhibit potential for practical applications in biomedicine, tissue engineering, and ecofriendly materials. Additional investigations on the modification of new substituents on ChNFs are expected to provide new gelling or soft materials with unique functions in the future.

## Data Availability

Not applicable.
